# Comparative Economic Analysis Between Endogenous and Recombinant Production of Hyaluronic Acid

**DOI:** 10.3389/fbioe.2021.680278

**Published:** 2021-07-21

**Authors:** Mario A. Torres-Acosta, Héctor M. Castaneda-Aponte, Liliana M. Mora-Galvez, Monica R. Gil-Garzon, Martin P. Banda-Magaña, Esteban Marcellin, Karla Mayolo-Deloisa, Cuauhtemoc Licona-Cassani

**Affiliations:** ^1^ The Advanced Centre for Biochemical Engineering, Department of Biochemical Engineering, University College London, London, United Kingdom; ^2^ Tecnológico de Monterrey, Escuela de Ingeniería y Ciencias, Monterrey, Mexico; ^3^ Núcleo de Innovación de Sistemas Biológicos, Centro de Biotecnología FEMSA, Tecnológico de Monterrey, Monterrey, Mexico; ^4^ Biomentum SAPI de CV, Guadalajara, Mexico; ^5^ Australian Institute for Bioengineering and Nanotechnology, The University of Queensland, Brisbane, QLD, Australia; ^6^ The Queensland Node of Metabolomics Australia, The University of Queensland, Brisbane, QLD, Australia

**Keywords:** hyaluronic acid, techno-economic analysis, recombinant production, endogenous production, cost of goods

## Abstract

Hyaluronic acid (HA) is a biopolymer with a wide range of applications, mainly in the cosmetic and pharmaceutical sectors. Typical industrial-scale production utilizes organisms that generate HA during their developmental cycle, such as *Streptococcus equi* sub. *zooepidemicus*. However, a significant disadvantage of using *Streptococcus equi* sub. *zooepidemicus* is that it is a zoonotic pathogen, which use at industrial scale can create several risks. This creates opportunities for heterologous, or recombinant, production of HA. At an industrial scale, the recovery and purification of HA follow a series of precipitation and filtration steps. Current recombinant approaches are developing promising alternatives, although their industrial implementation has yet to be adequately assessed. The present study aims to create a theoretical framework to forecast the advantages and disadvantages of endogenous and recombinant strains in production with the same downstream strategy. The analyses included a selection of the best cost-related recombinant and endogenous production strategies, followed by a sensitivity analysis of different production variables in order to identify the three most critical parameters. Then, all variables were analyzed by varying them simultaneously and employing multiple linear regression. Results indicate that, regardless of HA source, production titer, recovery yield and bioreactor scale are the parameters that affect production costs the most. Current results indicate that recombinant production needs to improve current titer at least 2-fold in order to compete with costs of endogenous production. This study serves as a platform to inform decision-making for future developments and improvements in the recombinant production of HA.

## Introduction

Hyaluronic Acid (HA) is the main component of the extracellular matrix and it regulates different skin-related signaling processes such as inflammation, cellular migration and angiogenesis, which are the main phases of tissue regeneration (Salwowska et al., 2016). HA is a biomolecule with a wide range of biological uses and potential industrial applications (Papakonstantinou et al., 2012; Bukhari et al., 2018; Bowman et al., 2018), particularly, it is widely used as a component of many commercial skincare and anti-ageing products. HA has broad consumer acceptance and its market was measured at USD 5.32 billion in 2012 and was expected to be USD 9.85 billion in 2019, with a market price of between USD 1,000 to 5,000 per kg depending on purity and the molecular weight of the polymer (de Oliveira et al., 2016; Marcellin et al., 2014).

The first process for HA production involved extraction from animal waste. Apart from ecologically unfriendly, this procedure is hampered by the inevitable degradation of hyaluronan, caused by endogenous hyaluronidase activity and the harsh conditions of extraction. As a result, microbial HA production emerged during the early 1980s as an alternative production platform as one of the first biotechnological alternatives for fine chemicals production. Current production of HA typically involves the use of *Streptococcus* equi sub. zooepidemicus (Salwowska et al., 2016; Papakonstantinou et al., 2012; Bukhari et al., 2018; Bowman et al., 2018; de Oliveira et al., 2016; Liu et al., 2011; Chong et al., 2005; Liu et al., 2008; Jeong et al., 2014; Jia et al., 2013; Chahuki et al., 2019). This microorganism has been reported to have high production yields (5–10 g/L), but its main disadvantage lies in its pathogenic nature (Liu et al., 2011). Recombinant production has emerged as a safer alternative since it poses no potential risks to operators or consumers. Reported recombinant organisms for HA production include Pichia pastoris (Jeong et al., 2014), *Lactobacillus* (Chahuki et al., 2019), and *Bacillus* subtilis (Jia et al., 2013), with the former being classified as “Generally Recommended as Safe (GRAS)” by the Food and Drug Administration (FDA) (Sewalt et al., 2016; Elshaghabee et al., 2017). The disadvantages of recombinant HA production include low production yields and the genetic instability of producing strains (Liu et al., 2011). While some recombinant HA producing strains achieve up to 6.8 g/L and have the possibility to control the HA molecular weight (Liu et al., 2011), endogenous organisms have the potential to produce more HA in a shorter time (Liu et al., 2011). On the other hand, HA downstream bioprocessing has been extensively tested. The vast majority of the methods involved rely on a series of precipitations, filtrations, liquid-liquid partitions or chromatographic operations (Cavalcanti et al., 2019; Sousa et al., 2009; Choi et al., 2014; Rajendran et al., 2016; Akdamar et al., 2009; Murado et al., 2012; Rangaswamy and Jain, 2008), each of these with specific variations to reduce material consumption or increase recovery yields.

Novel platforms for HA production and recovery/purification raise questions in terms of applicability in an industrial setting. Appropriate evaluation prior to implementation can provide useful insights towards decision-making and to efficiently distribute available resources to achieve production at large scales. Bioprocess modeling and techno-economic analyses (TEA) are powerful tools that help with decision-making prior to the design of a bioprocess (Heinzle et al., 2006). This ultimately seeks to address where resources and optimization efforts should be focused on. TEAs form part of most bioprocess applications from their conception to implementation in industrial settings (Heinzle et al., 2006). For example, they have been successfully used in a wide range of biotechnological applications, such as contrasting production of monoclonal antibodies in stainless steel or single-use technologies (Farid et al., 2005), fed-batch and perfusion fermentation alternatives (Lim et al., 2005; Lim et al., 2006), examination of the costs associated with the generation of neutrophils for neutropenic patients (Torres-Acosta et al., 2019a), or selection among purification techniques (chromatography or aqueous-two phase systems) for a pharmaceutical enzyme (Torres-Acosta et al., 2015), among others. Moreover, the use of computational tools allows the creation of a diverse array of potential production scenarios to determine the robustness of a model or to identify critical parts in a process (Heinzle et al., 2006; Torres-Acosta et al., 2019b). This can ultimately contribute to both decision-making and efficient resource allocation.

In the present study, we report a theoretical framework for the economic assessment of endogenous and recombinant production of HA. First, the upstream processing section is analyzed and the best options from both sources selected. These are then integrated with the downstream processing into a complete bioprocess, based on reported data, in order to reflect the full costs of production. A sensitivity analysis is performed to determine which process parameters have the highest impact on both production settings. A series of Monte Carlo simulations are then performed on the most critical parameters to be varied simultaneously. Finally, assuming endogenous production as a gold standard, our results aim to estimate the minimum HA yield required through recombinant production in order to obtain similar production costs obtained with endogenous production. This study serves as a platform that will aid decision-making for future developments in HA production and its downstream processing.

## Model Set-Up

The model was constructed using Biosolve Process (Biopharm Services Ltd., United Kingdom). The approach employed in this study consisted of using data obtained from the literature to evaluate a variety of techniques reported for endogenous and recombinant production. These were then incorporated into a complete bioprocess to obtain insights into the behavior of production costs and to serve as a framework for decision-making. This study focused exclusively on cost related directly with production. Costs related with further packaging, storage, distribution, etc., were not considered. Additionally, it is critical to visualize that endogenous production utilizing *Streptococcus* equi sub. zooepidemicus will have increased production costs to the ones calculated here due to the requirement to operate inside a GMP facility. On the other hand, recombinant production will also require clinical trials to determine the safety of the product. The purpose of this study is to focus on production costs regarding only unit operations and production yields to determine which option can potentially generate lower costs and justify a future investment.

Currently, there are approaches for generating HA as a potential product from endogenous (Liu et al., 2008; Rangaswamy and Jain, 2008; Zhang et al., 2006) and heterologous (or recombinant) (Jeong et al., 2014; Jia et al., 2013; Yoshimura et al., 2015) sources. It is important to note that the published studies that use endogenous production for potential scale-up utilize the pathogenic bacteria *Streptococcus* equi sub. zooepidemicus, while recombinant productions use a range of different organisms. An economic model was constructed based on a literature review of reports for upstream (HA production) and downstream (HA recovery and purification) processing (Table 1). The output of this model consists of the production costs, which were expressed as production costs per gram of HA at the end of the complete process (CoG/g), or production costs per batch (CoG/batch).

**TABLE 1:** Data from hyaluronic (HA) acid upstream processing reports used for model construction. Data includes HA source, production results, reported conditions and fermentation media.

Hyaluronic acid source	Production option	Organism (strain)	Hyaluronic acid production	Reported conditions	Fermentation media (g L^−1^)
Endogenous production	Option 1 Zhang et al. (2006)	*Streptococcus equi* sub. *Zooepidemicus* (NJUST01)	6.7 g L^−1^ >1.5 MDa	Batch 36 h220 rpm/37°C	Starch—20 Glucose—50Peptone—3MgSO_4_—5K_2_HPO_4_—1.5
	Option 2 Liu et al. (2008)	*Streptococcus equi* sub. *Zooepidemicus* (WSH-4)	6.6 g L^−1^	Fed-batch/Batch8 h/20 h 200 rpm/37°C/0.5 vvm Fed-batch sucrose feed at 1g L^−1^; batch sucrose initial conc. 15 g L^−1^	Sucrose—75 Yeast extract—25 K_2_SO_4_—1.3 MgSO_4_-7H2O—2 Na_2_HPO_4_-12H_2_O—6.2FeSO_4_-7H_2_O—0.005 ZnCl_2_—0.00012 CuSO_4_·5H_2_O—4.75 × 10^−5^
	Option 3 Rangaswamy and Jain (2008)	*Streptococcus equi* sub. *Zooepidemicus* (ATCC 39920)	5 g L^−1^ 4 MDa	Batch 24 h 400 rpm/37°C/4 vvm	
					Sucrose—50 Yeast extract—3.5 K_2_HPO_4_—2 Casein—25 NaCl—1.5 MgSO_4_·7H_2_O—0.4
Recombinant production	Option 4 Yoshimura et al. (2015)	*Streptomyces albulus*	6 g L^−1^ 3 MDa	Batch 72 h 500 rpm/30°C/3.5 vvm	Glucose—50 (NH_4_)_2_SO_4_—1 Na_2_HPO_4_—1.6 KH_2_PO_4_—1.4 MgSO_4_·7H_2_O—0.5 ZNSO_4_·7H_2_O—0.04 FeSO_4_·7H_2_O—0.03
	Option 5 Jia et al. (2013)	*Bacillus subtilis*	6.8 g L^−1^ 4.8 MDa	Fed-batch 48 h 200 rpm/37°C Induction with IPTG (1 mM) and xylose (2% w/v)	Yeast extract—5 Peptone—10 NaCl—10
	Option 6 Jeong et al. (2014)	*Pichia pastoris*	0.76 g L^−1^ 2.5 MDa	Fed-batch 48 h 500 rpm/30°C (growth)/26°C (production)/0.7 vvm, pH 7 Induction with methanol at 0.5% v/v	Glucose—40 Yeast extract—7.5 Peptone—10 K_2_HPO_4_—2.5 MgSO_4_—0.5 NaCl—5

For upstream processing (HA production), the literature review targeted a collection of results and protocols of HA production with promising results (high production titer) and HA of high molecular weight (main component of high-end HA products). A total of six reports were selected (three for endogenous and three for recombinant production) and are presented in Table 1. Briefly, for endogenous (*Streptococcus* equi sub. zooepidemicus) production, these studies presented production yields that ranged from 5 to 6.7 g/L, with a fermentation lasting 20–36 h. For recombinant production, the yields ranged from 1.6 to 6.8 g/L, with a fermentation duration of 48–72 h.

For downstream processing (HA recovery and purification), a report based mainly on precipitation and filtration (Rangaswamy and Jain, 2008) was selected for modeling HA recovery from both productions (endogenous and recombinant), since it was assumed that this process can achieve similar HA purification yields regardless of the HA source. This assumption must be verified by laboratory research but, for modeling applications, it can be utilized to determine economic behavior. Briefly, following HA production, the fermentation broth is diluted with water and centrifuged to remove biomass. Isopropanol is added to the supernatant to induce HA precipitation and this is centrifuged once again for collection. Precipitated HA is then dissolved with a sodium acetate solution and silica gel added for further contaminant removal, with a third centrifugal step included for clarification. The supernatant is then processed through an activated carbon filter, followed by a diafiltration step for concentration and buffer exchange. The process is completed with a 0.22 µm filtration to obtain a sterile product. Details of relevance to the economic model of the bioprocess are presented in Table 2. A diagram has been included in Supplementary Material for visual representation of the process designed here.

**TABLE 2:** Data from hyaluronic (HA) acid downstream processing reports used for model construction. Unit operations were adjusted for duration to be 1 h each (Preparation + Process + Cleaning). Unit operation one corresponds to fermentation (explained in Table 1). Letter X, Y, Z, and C were used in substitution of the actual value of recovery yield, centrifuge flow rate, filtration area, and HA concentration, respectively. Actual values for these parameters vary according to the variables analyzed in this study.

	Unit operation	Equipment	Recovery yield	Economic-relevant process parameters
Unit operation 2	Dilution with water	Stainless steel stirred tank	95%	Water addition 1:1
				C^a^ HA g L^−1^
				1 h
Unit operation 3	Biomass removal	Centrifuge	90%	Y^b^ L h^−1^
				1 h
Unit operation 4	Mixing with isopropanol (induction of HA precipitation)	Stainless steel stirred tank	X^c^%	Isopropanol addition 1:1
				C^a^ HA g L^−1^
				1 h
Unit operation 5	Precipitate recovery	Centrifuge	90%	Y^b^ L h^−1^
				1 h
Unit operation 6	Resuspension with sodium acetate	Stainless steel stirred tank	X^c^%	[NaAc]: 30 g L^−1^
				C^a^ HA g L^−1^
				1 h
Unit operation 7	Removal of contaminants with silica gel	Stainless steel stirred tank	X^c^%	Silica gel: 3% w/v
				C^a^ HA g L^−1^
				1 h
Unit operation 8	Removal of silica gel	Centrifuge	90%	Y^b^ L h^−1^ 1 h
Unit operation 9	Activated carbon filtration	Flow-through filtration	X^c^%	C^a^ HA g L^−1^
				Z^d^ m^2^
				4 L m2^−1^ h^−1^
				1 h
Unit operation 10	Ultrafiltration and diafiltration	Tangential ultrafiltration	X^c^%	C^a^ HA g L^−1^
				Z^d^ m^2^
				4 L m2^−1^ h^−1^
				1X concentration factor
				5 diavolumes
				1 h
Unit operation 11	Sterility filtration	Flow-through filtration	95%	C^a^ HA g L^−1^
				Z^d^ m^2^
				4 L m2^−1^ h^−1^
				1 h

c Recovery yield for this unit operations was changed between 20, 40, 60, 80, and 100% to understand its impact on the overall production costs and CoG/g. All combinations were studied and included in this work results.

b Flow rate of centrifugal operation was adjusted to maintain total operation time of 1 h. $Flow\;Rate\;\left[L\;h^{-1}\right]=\frac{Volume\;In\;\left[L\right]}{Operation\;Time\;\left[1\;h\right]}$

d Membrane size (m2) was calculated by using a constant flux of 4 L m2-1 h-1 and a process time of 1 h. $Membrane\;Size\;\left[\;m^{2}\right]=\frac{Volume\;In\;\left[L\right]*Process\;Time\;\left[1\;h\right]}{Operation\;Time\;\left[4\;L\;m^{2\;-1}\;h^{-1}\right]}$

a HA concentration varied according to the operation scale and recovery yield being analyzed. $HA\;Concentration\;\left[g\;L^{-1}\right]=\frac{Volume\;In\;\left[L\right]*HA\;Concentration\;from\;previous\;operation\;\left[g\;L^{-1}\right]*Recovery\;Yield\;\left[\%\right]}{Volume\;Out\;\left[L\right]}$

### Model Construction

Regardless of the upstream or downstream selection, the model must include three major areas (Torres-Acosta et al., 2019b): 1) production scenarios for evaluation, 2) unit operation and process parameters, and 3) the economic dataset that will populate the model. It is important to note that this study focuses only on the production process and not on commercial aspects, such as transport, storage, and commercialization.

Since this study is solely based on reports from other authors, the model was constructed using their data for building production scenarios, with the flexibility to incorporate wide ranges to evaluate the impact of different variables on production costs. For this reason, the variables for evaluation included production titer, capital cost and operation times. These three variables were modified by multipliers from their reported (titer and operation times) or calculated (capital cost) base. These multipliers were 0.1X, 0.5X, 1X, 2X and 10X times the original value (a range from ten times higher to ten times lower than the base). Other variables with different modifiers were included: bioreactor scale (1, 10, 50, 100, 250, 500 and 1,000 L), recovery yield of a set of selected unit operations (20, 40, 60, 80 and 100% of efficiency) and a discount on material costs (0, 20, 40, 60 and 80%) (Table 2). Considering the multipliers shown here and particularly for the production titer, this will mean an approximate range from 0.68 to 68 g/L. The maximum titer of 68 g/L has not been reported and can be practically impossible to achieve. Regardless of the practical constrains, having a wide range allows to obtain data to generate to visualize complete scenarios and determine interesting results that will help to determine if it is viable to invest on a recombinant source.

For the second area of the model, and for simplicity, the upstream portion was analyzed first. The least expensive option for both the endogenous and recombinant production was then selected and incorporated into the complete process (upstream plus downstream processing). This bioprocess contains eleven-unit operations, of which six had a fixed recovery yield given by the Biosolve Process default settings, while the values of the five remaining operations were varied among those already presented. This decision was taken because unit operations with fixed yield are common for a range of bioprocesses, while those with a variable yield are specific to this process. All of the process parameters and unit operations are presented in Table 1 (upstream section) and Table 2 (downstream processing).

The third pillar for model creation is that of the economic datasets. This set of information will populate the model with economic value to obtain the production costs and it comprises four main areas: capital, materials, consumables, and labor (detailed information is presented in Table 3). For this study, the term capital considers the cost of equipment acquisition and facility construction. This information was obtained directly from the Biosolve Process database. Equipment cost was obtained at specific scales and regressions performed in order to interpolate values. For facility construction, this study employed a range of factors that correlate every aspect of the construction with the capital invested in equipment acquisition (automatically calculated by Biosolve Process). In this study, materials are related to fermentation media and HA recovery/purification, and their costs were obtained from Sigma-Aldrich, assuming purchase of the largest presentation available (which correlated to the least expensive price per unit of mass). This approach allowed an overestimation of the material costs and, in order to stay above the real cost and prevent underestimation (Torres-Acosta et al., 2020), a discount was also included in the analysis in order to reflect the reduction in prices for bulk acquisition implicit in the larger scales analyzed here. Consumables are primarily related to filtration steps and their costs were obtained from the Biosolve Process database. Finally, labor has been studied previously (Heinzle et al., 2006; Torres-Acosta et al., 2021) and it is estimated to account for approximately 15% of the production costs, so this fixed value was adopted for the purposes of this study.

**TABLE 3:** Economic dataset employed to populate the model for HA production.

Item	Cost or cost/g (US $)	Rationale
	Equipment	
Bioreactor	$Bioreactor\;Cost\;\left[US\;\$\right]=34,854*Bioreactor\;Volume^{0.4058}$	*Biosolve Process database contains a collection of costs for all equipment at specific scales. These regressions were constructed in order to interpolate to all possible operation scales analyzed here*
Centrifuge	$Centrifuge\;Cost\;\left[US\;\$\right]=42,6720*{\left(\frac{Centrifuge\;Volume}{600}\right)}^{0.4}$	
Stainless steel stirred tank	Tank Cost [US $]=42.195∗Tank Volume [L]+3,052.2	
Filtration (flow-through)	$Equipment\;Cost\;\left[US\;\$\right]=2,229.6*Membrane\;Area^{0.4539}$	
Filtration (tangential ultrafiltration)	$Equipment\;Cost\;\left[US\;\$\right]=91,036*Membrane\;Area^{0.3741}$	
**Materials (US $ *per* kg)**		
Glucose	*10.3*	*As explained in text, prices were obtained at from the largest available presentation from Sigma-Aldrich (St. Louis, MO, USA). Subsequently, this prices were discounted accordingly to the analysis conducted in this work*
Peptone	*51.2*	
MgSO**4**	*36.8*	
MgSO**4**-7H**2**O	*40.8*	
K**2**HPO**4**	*110.7*	
Starch	*16.6*	
Sucrose	*16.8*	
Yeast extract	*55.9*	
K**2**SO**4**	*57.6*	
Na**2**HPO**4**	*31.4*	
FeSO**4**-7H**2**O	*92.6*	
CaCl**2**	*63.7*	
ZnCl**2**	*27.3*	
CuSO**4**-5H**2**O	*54.7*	
Casein enzyme hydrolysate	*143.9*	
NaCl	*10.9*	
Isopropanol	*20.8*	
Sodium acetate	*21.0*	
(NH**4**)2SO**4**	*17.4*	
KH**2**PO**4**	*32.9*	
ZnSO**4**-7H**2**O	*104.2*	
Silica gel	*17.0*	
**Consumables**		
Vessel filters (used before entry into a vessel)	Vessel Filter Cost (US $)=0.3058∗Vessel Volume (L)+45.334	*Costs obtained from Biosolve Process database*
Activated carbon filter	Automatically adjusted by biosolve process when changing production scale base cost: US $575 for 1 m^2^	
Ultrafiltration membrane	Automatically adjusted by biosolve process when changing production scale base cost: US $4,621 for 1 m^2^	

### Comparative Analyses Between Recombinant and Endogenous Hyaluronic Acid Production

The present study is divided into three parts. The first analysis was a direct comparison between the recombinant and endogenous HA production scenarios–this allowed selection of the best of each approach. Since the only unit operation analyzed is that of the production stage (upstream process), the cost calculation, and thus the contrast, is heavily focused on the costs of consumables and materials, fermentation titer, and process time. This means that the costs of capital investment (equipment and facility construction) were not considered in this stage. The model was subsequently completed with the selected production settings (full process considering full costs), and a sensitivity analysis performed (Lim et al., 2005; Lim et al., 2006; Farid, 2007). For this, each variable was analyzed individually and systematically. This bioprocess comprises eleven unit operations, five of which were varied between 20 and 100% of yield (overall yield ranged from 0.02 to 66% of HA at the end of the process). Moreover, it was decided to analyze the fermentation titer, scale, capital, materials discount and fermentation times for all the possible overall recovery yields calculated. All of the possible combinations, along with their results, are included in Supplementary Material. This sensitivity analysis allowed determination of the impact of each variable on the CoG/g, after which the three variables with the highest contribution were varied randomly (Monte Carlo simulations) and simultaneously in order to obtain the overall possible range of CoG/g under the scenarios created (Farid, 2007). To summarize this data, a multiple linear regression was constructed in the software R (Torres-Acosta et al., 2020). A final comparison of the two production sources was also performed to determine the scenarios under which recombinant production could be less expensive than endogenous HA production.

## Results and Discussion

Hyaluronic acid is a biopolymer utilized in different current sectors of biotechnology (Papakonstantinou et al., 2012; Bukhari et al., 2018; Bowman et al., 2018), and decreasing its production costs is important. Multiple reports for improvements in the production (Liu et al., 2011) and recovery/purification (Cavalcanti et al., 2019; Sousa et al., 2009; Choi et al., 2014; Rajendran et al., 2016; Akdamar et al., 2009; Murado et al., 2012; Rangaswamy and Jain, 2008) exist. However, a theoretical framework is required in order to determine which options provide the best production costs and have the potential to be incorporated into real-life production. The use of a model can also help to determine potential areas of opportunity that might be key for further improvements. This can translate into a reduced requirement for investment of resources and more intelligent decision-making.

### Analysis of Endogenous and Recombinant Hyaluronic Acid Production (Upstream Processing)

**FIGURE 1:**
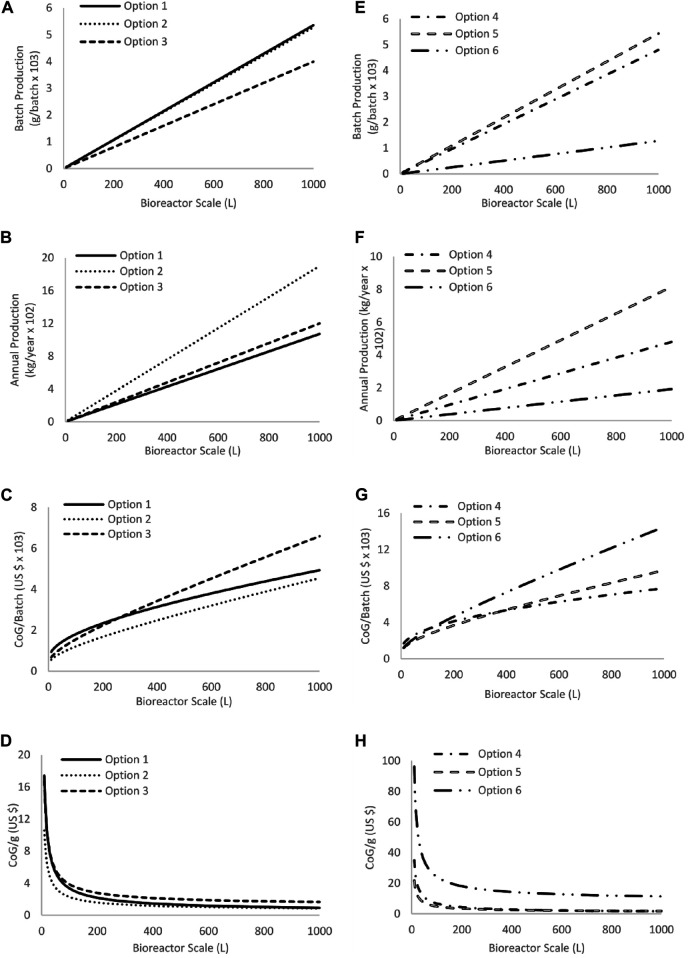
Results contrasting fermentation options for endogenous and recombinant production. Specific conditions of each option (1–6) are presented in Table 1. **(A–D)** show results for endogenous production and **(E–H)** for recombinant production. panel 1A,E present batch production in grams per batch, panel 1B,F results for annual production (kg per year), **(C,G)** the cost of production per batch (US $ x103), and Figures 1D,H results for CoG/g (US $). All results were calculated for the bioreactor scales from 1 to 1,000 L in steps of 1 L.

First, we compared endogenous (*Streptococcus* equi sub. zooepidemicus) and recombinant production in terms of HA molecular weight and yield, fermentation setting and culture media utilized (Production scenarios are presented in Table 1 and the economic data in Table 3). For readability, each HA production option will be denoted as option one to option 6, accordingly to Table 1 (options one to three for endogenous and 4–6 for recombinant production). The results for each type of production are presented in Figure 1. This initial analysis was performed by utilizing base scenarios (reported fermentation titer and operation time) for each option, while varying the scale of production (1–1,000 L).

The results for endogenous production indicate that, as expected, the options that generate a greater output of product imply a lower production cost (either CoG/g or CoG/batch). One of the main factors that affect these results is operation time. Given that the only unit operation in this first stage (production analysis) is the fermentation, the operation time relates to the duration of production. Figure 1A–F represent the mass that can be generated in every batch and the potential annual production (considering 200 operational days per year). The most interesting result is option 1, which shows the highest amount per batch but the lowest amount per year. This is because option 1 has the highest production titer (6.7 g/L) but also the longest production time (36 h). Alternatively, option 2 has the second-highest yield (6.6 g/L) but the lowest production time (20 h). For this reason, option two was selected as the best endogenous HA production setting. It is important to note that option 1 has the highest CoG/batch at low production scale, but this decreases to become the second most expensive as scale increases, which is explained by the cost of materials. Option 1 has the least expensive media from the three options, giving its contribution less impact as batch size increases (the cost per batch for materials presents a linear increase with increasing batch size, i.e., double the size equals double the cost for materials). This stabilizes the CoG/batch, while options two and three continue to increase since they involve more expensive materials.

For recombinant production, the behavior of overall production costs is the same as for endogenous production, although it is interesting that option 4 has the lowest CoG/batch at large scales but not at a small scale. The decision regarding which is the best recombinant option is therefore not a straightforward one. For potential commercial implementation, annual production and product per batch are important parameters to consider since they will translate into the actual product available for sale. Option 5 has the lowest CoG/batch at small scales (below 381 L), but option 4 has the same result at large scales (above 381 L). Alternatively, option 5 has the largest batch and annual production since it has the largest production titer and lowest fermentation time. Also, the difference in CoG/g between options four and 5 is relatively small. For these reasons, option 5 was selected for further analysis.

### Sensitivity Analysis

**FIGURE 2:**
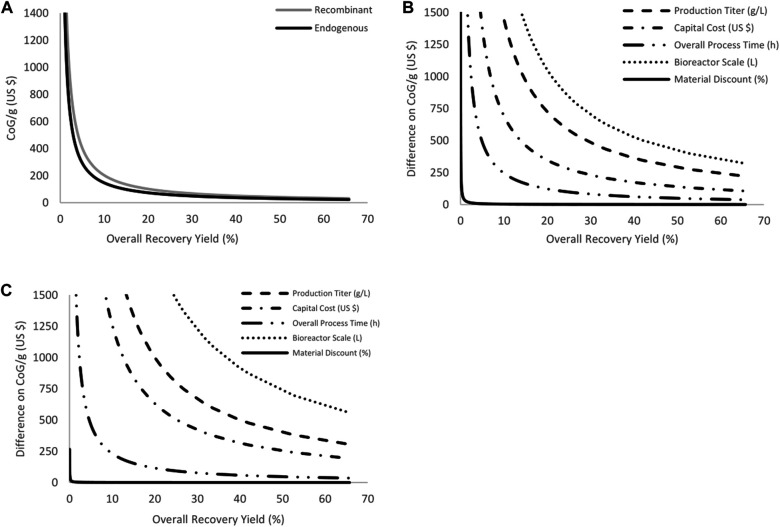
Results from the sensitivity analysis performed on endogenous and recombinant production. Variables analyzed comprise production titer, capital costs, overall process time, bioreactor scales, materials discount and overall recovery yield. **(A)** presents basal results for both production options at all recovery yields evaluated when the remaining variables are at 1X, operating at 100 L with 0% materials discount. **(B)** (endogenous) and **(C)** (recombinant) shows the difference between the maximum and minimal CoG/g when each variable was analyzed.

Following the selection of options 2 and 5, the model was completed as described above. The downstream processing portion of the bioprocess was added and a selection of variables analyzed. Each parameter was varied individually and systematically according to the ranges presented in the previous section. Since the recovery yield was varied between five values (100, 80, 60, 40 and 20%) for a selection of five unit operations, this generated 3,125 possible combinations (Representative results for endogenous and recombinant production at all of the recovery yields at basal conditions - 1X multipliers for variables, 100 L bioreactor scale and 0% material discount - are presented in Figure 2A). It was decided to look at the variations for the rest of the variables (fermentation titer, capital cost, overall process time, production scale and materials discount) at each of the scenarios for the overall recovery yield, which ranged from approximately 0.02–66%. The analysisgenerated 84,375 different possibilities, for which the CoG/g values obtained are summarized in Figure 2B (endogenous production) and 2c (recombinant production), while full results are included in Supplementary Material; Figure 2B,C show the difference between the highest and lowest CoG/g obtained by modifying each variable between the ranges presented (i.e., in analysis of the production titer, this is the CoG/g at 0.1X minus the CoG/g at 10X). For the purposes of comparison (and selection for the next analysis), at a given overall recovery yield, the higher the value shown in the graphs of Figure 2B,C, the higher the contribution of that variable to the CoG/g. For example, the bioreactor scale has the highest value from all of the variables, meaning that this parameter has the strongest effect on the CoG/g. This indicates that variation, i.e., increasing or decreasing the value, of any given variable can have a profound effect on the CoG/g. This phenomenon shows that variables with the highest impact should be controlled during production in order to minimize production cost or reduce potential variability. It should be noted that the base conditions considered for the sensitivity analysis are at the 1X multiplier (along with using a 100 L scale and 0% materials discount) using the base values presented in Table 1 for upstream, Table 2 for downstream, and Table 3 for economic datasets.

The overall ranking of impact on the CoG/g is the same for endogenous and recombinant production. The most critical parameter is production scale, which has been reported previously as one of the major contributors affecting the CoG/g in other bioprocesses (Torres-Acosta et al., 2019a; Pollock et al., 2013). This is caused by the effect that changing scale has on product generation, which is described as having a linear behavior (Figure 1A). CoG/batch increases when production scale increases, but one of the major contributors to this is the capital investment. The cost of equipment acquisition generally rises with increasing size; however, at large scale, this cost tends to stabilize (i.e., the cost difference between a 10 L and a 20 L bioreactor is proportionally greater than the difference between a 110 L and a 120 L bioreactor), causing the impact of production scale to be highly significant.

The variable with the second-highest impact on cost is the fermentation titer. This has also been reported as a parameter to which bioprocess costs are highly sensitive (Torres-Acosta et al., 2021; Farid, 2007). It is important to develop strategies to optimize fermentation titer by increasing its yield or by decreasing its variability between batches. The reason for its importance is similar to that of the scale of operation explained above. As the fermentation titer increases, the amount of product also increases, which dilutes the costs by the amount of product being generated. The rest of the variables have a lesser impact, but still merit analysis. The capital cost, overall process time and materials discount do have an important effect on the CoG/g, but their effect is lower than that of bioreactor scale and fermentation titer since they do not directly affect the amount of product being generated. The materials discount can be seen to rapidly lose impact. The results for materials discount support the decision to use small-scale prices while including a discount in the analysis. This means that, even at large scales, expensive materials have a lower impact compared to the rest of the variables analyzed here.

Another parameter studied indirectly, although critical for CoG/g behavior, is that of overall recovery yield. From Figure 2 (particularly Figure 2A), recovery yield is the variable plotted in the *x*-axis and it can be seen to have an impact on the CoG/g for all of the variables studied. As overall recovery yield increases, CoG/g sharply decreases and tends to stabilize at high recovery yields. This translates into an important effect in terms of the direct contribution of this variable to the product generated. Furthermore, its stabilizing effect on CoG/g behavior for other variables indicates that this could be the most important variable. For this reason, bioreactor scale, production titer and overall recovery yield were selected for inclusion in a subsequent analysis in which these variables were simultaneously evaluated (Monte Carlo simulations).

**FIGURE 3:**
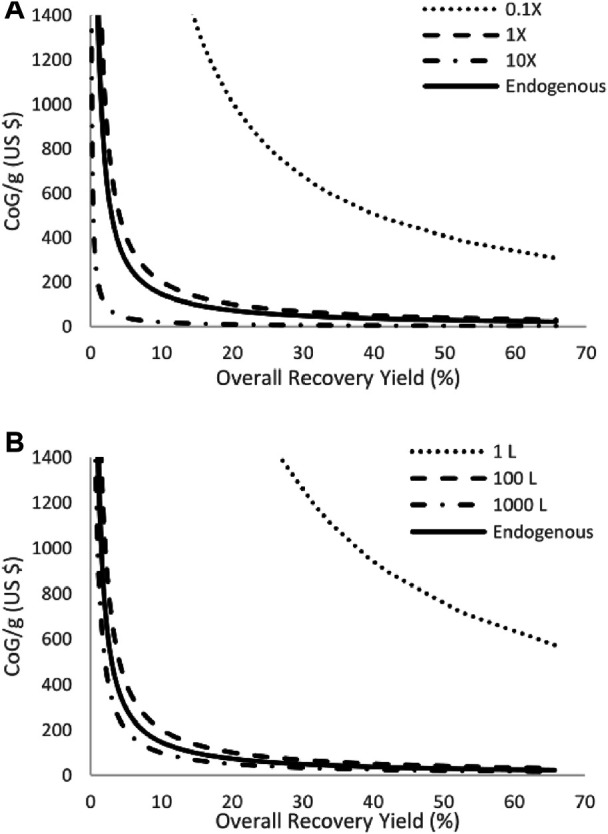
Individual sensitivity analysis results for production titer **(A)** and bioreactor scale **(B)** at all the recovery yields analyzed. Results show a possible lower CoG/g for titer when optimizing this parameter. Alternatively, bioreactor scale can achieve higher CoG/g when reducing its operation size.

Another aspect for consideration regarding production titer and bioreactor scale, beyond the difference between the highest and lowest CoG/g (Figure 2B,C), is the effect on CoG/g as a result of their individual variation. This is achieved by showing how low the CoG/g is when contrasting the 1X and 10X scenarios for production titer and the 100 L and 1,000 L for bioreactor scale. Figure 3 shows this comparison for recombinant production only since the endogenous production behaves equally. Figure 3A shows the results for bioreactor scale and Figure 3B for the production titer. The results in Figure 3 indicate that the behavior of each variable is different when deviating from the 1X or 100 L scenarios. From these results, it is evident that production titer can achieve a lower production cost by increasing to its 10X scenario, than by moving to a 1,000 L bioreactor scale. However, this behavior is inverted when both parameters decrease 10-fold (0.1 X scenario for production titer or 1 L for bioreactor scale). From the results in Figure 2, the overall contribution to the CoG/g is larger from the bioreactor scale contribution but, for future practical implementation, it is better to achieve lower CoG/g.

For this reason, it could be concluded that fermentation titer makes an important contribution since it can decrease the CoG/g by being optimized. It is important to note that the 10X scenario for fermentation titer is approximately 68 g/L (1X = 6.8 g/L), which would be unrealistic for actual HA production. The purpose of using such an extreme value is to develop a more profound understanding of the impact of this parameter on the CoG/g. Moreover, using drastic (unrealistic) values can help to estimate the extent that the value recombinant production should be optimized to generate a lower production cost. In a subsequent section, the actual range for the fermentation titer needed to generate a lower cost is further analyzed.

The sensitivity analysis allowed us to determine critical parameters but also to observe their behavior while obtaining different overall recovery yields. This is highly important because, prior to the practical implementation of a bioprocess, it is necessary to understand how sensitive the process is to variations of different parameters. This particular process for HA production should aim to operate at large scales, while controlling a high production titer and recovery yield. This is desirable for any given process since high production and recovery will always yield more product. However, it is important to consider that, in practice, it could be simpler to work on developments to obtain a higher production titer. Furthermore, overall recovery yield is a parameter that is dependent on the yield of each unit operation and, to obtain a high recovery, every operation is required to have a high yield. This can therefore become complicated, since some unit operations are already at their maximum (i.e., centrifugation operations), while others could require further research (i.e., isopropanol precipitation, sodium acetate solubilization, etc.). In contrast, the improvement of fermentation titer directly affects a single unit operation and has great potential for rapidly decreasing the CoG/g.

In summary, the variables with the highest impact are bioreactor scale, fermentation titer and overall recovery yield, although fermentation titer can generate a lower CoG/g than bioreactor scale at 1,000 L. Moreover, improvements in titer affect the process more rapidly by focusing its effect on a single operation, rather than on the overall recovery yield. All three variables directly affect the amount of product to be generated. As they increase, the overall costs are diluted by the quantity of product obtained. Recovery yield and fermentation titer can be optimized in the laboratory, while modifying the working scale is a more commercial decision, since increasing the size of the bioreactor will directly affect the CoG/batch and capital expense.

### Simultaneous Evaluation of Model Variables

An additional study was conducted to understand the impact of production titer, bioreactor scale and recovery yield. A series of Monte Carlo simulations (Torres-Acosta et al., 2020; Farid, 2007; Torres-Acosta et al., 2018) were performed in which the production titer and bioreactor scale were able to change randomly in the ranges of 0.1X to 10X multipliers and 1 L to 1,000 L, respectively. For each of the 175 combinations generated, simulations were run at all the overall recovery yields calculated (0.02–66%) and the CoG/g recorded in each case. The full results are presented in Supplementary Material. This analysis produced 546,875 (175 combinations between titer and scale multiplied by the 3,125 possible recovery yields) different values for CoG/g for the endogenous and another equal set for the recombinant production.

As expected, the results indicate that the lowest CoG/g for recombinant production ($1.53 USD) is only achieved under an unrealistic scenario of 10X the actual titer, when operating a bioreactor at a 1,000 L scale, with 66% overall recovery (the highest values for each of the variables). Although the titer required is much higher than that reported (Liu et al., 2011; Jia et al., 2013), this analysis serves to demonstrate the economic potential of improvements developed in the laboratory for both upstream and downstream bioprocess. More realistically, using this same approach, and given the same conditions, the lowest CoG/g for endogenous production is $1.38 USD. The difference between the two is much lower than at the base condition and, if recombinant production could actually decrease its cost to $1.53 USD per gram, it is approximately 20 times the CoG/g of endogenous production at its base scenario ($30.85 USD).

It is possible to summarize the entire data in a multiple linear regression, and it is important to note that the data does not present linear behavior. However, the regression coefficients for each variable indicate the “strength” that can be generated in the CoG/g by changes in that variable. The coefficients in a multiple linear regression can therefore help to elucidate the importance rank of these parameters, especially when they are varied simultaneously. The results of this regression are presented in Table 4. Using these results, the overall recovery yield for both production sources (recombinant and endogenous) can be seen to be the most important factor since it exhibits the largest coefficient (this was suggested in the previous section and now confirmed). This is followed by production titer and, finally, bioreactor scale. These results can be explained by the profound impact of recovery yield shown in Figure 2; as the value of this variable increases, it dominates all other parameters and decreases the CoG/g significantly to ultimately stabilize them at a low value (the exact stable value depends on the parameter in question). A counterintuitive result is that production titer has a larger coefficient than the bioreactor scale (Figure 2). This can be explained by the reasons presented above, in which production titer can achieve a lower CoG/g that, in turn, forces the coefficient to increase.

**TABLE 4:** Results for linear multiple regression.

Regression parameters^a^	Endogenous	Recombinant
	Coefficient	Coefficient
Intercept (β0)	1.871 × 10^3^ ^b^	2.477 × 10^3^ ^b^
Production titer (g/L) (β1)	−2.220 × 10^2^ ^b^	−2.824 × 10^2^ ^b^
Overall recovery yield (%) (β2)	−5.746 × 10^3^ ^b^	−7.318 × 10^3^ ^b^
Bioreactor scale (L) (β3)	−3.338 × 10^−1^ ^b^	−7.009 × 10^−1^ ^b^

a Regression with the form CoG/g [US $] = *β*0 + *β*1 x Production Titer [g/L] + *β*3 x Overall Recovery Yield [%] + *β*3 x Bioreactor Scale [L].

b Statistically significant to *α* = 0.01.

The results presented here are in agreement with reports in which fermentation titer, recovery yield and production scale (Torres-Acosta et al., 2015; Torres-Acosta et al., 2021; Farid, 2007; Rosa et al., 2011) are considered key parameters of bioprocess costs. Future endeavors in HA production should focus on increasing the production process to larger scales, while having a high production titer. It is recommended to achieve a high recovery yield but, given the difficulty of improving the overall yield (conjunction of individual unit operation yields), a suggestion could be made to improve the unit operation with the lowest recovery yield in the bioprocess.

### Comparison Between Endogenous and Recombinant Production

As demonstrated in the previous sections, endogenous production under the same conditions (of bioreactor scale, recovery yield and their respective production titers) is less expensive than recombinant production. These results favor future developments to use endogenous organisms, namely *Streptococcus* equi sub. zooepidemicus. Although this could be seen as a disadvantage to recombinant production, it also creates an area of opportunity for future research. Endogenous production using *Streptococcus* equi sub. zooepidemicus is considered potentially dangerous, given its pathogenic nature (Liu et al., 2011). Actual implementation will require the consideration of a large biosafety installation, which will increase production costs, particularly in terms of capital investment and facility construction time, that are beyond the costs analyzed here and the scope of this study. Moreover, consumables related to biosafety rather than production must also be considered. For this reason, recombinant production, particularly that of option four studied here, can rely on HA generation using *Bacillus* subtilis, which is considered an organism “Generally Recommended as Safe (GRAS)” by the Federal and Drug Administration (Sewalt et al., 2016; Elshaghabee et al., 2017).

**FIGURE 4:**
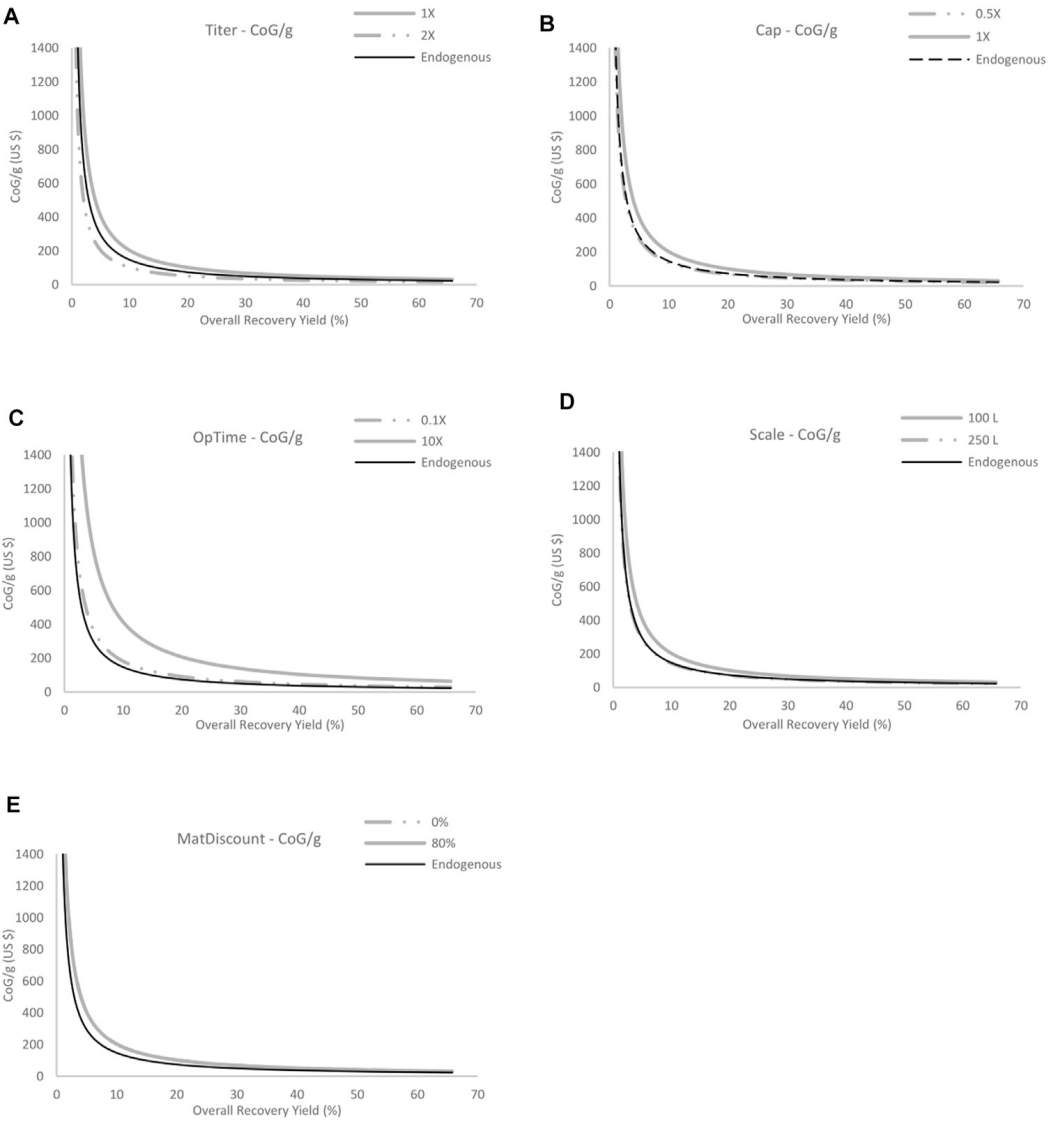
Contrast of basal endogenous production (100 L, 0% material discount, 1X multipler for the rest of the variables) and recombinant production at each individual variable analyzed here–Production titer **(A)**, capital costs **(B)**, overall operation time **(C)**, bioreactor scale **(D)**, and materials costs discount (E). Data shown includes only the scenarios that flank endogenous CoG/g. For the variables operation time and materials discount as they are never below endogenous CoG/g, the maximum and minimum CoG/g are presented. For bioreactor scale **(D)** the endogenous and 250 L results are close to each other and difference is almost negligible. For materials discount **(E)**, the 0 and 80% difference is almost negligible and appear almost overlapped.

Using the results from the sensitivity analysis, a new approach was implemented to envision how much improvement will be required by each of the variables analyzed here (production titer, bioreactor scale, overall recovery yield, materials cost discount and capital costs) (Figure 4). This set of graphs shows the CoG/g for endogenous production at the base scenario (100 L with 1X multipliers for all the variables) and at the two scenarios for recombinant production that flank (one more expensive and one less expensive) the CoG/g for the endogenous option. For those variables that, at any given multiplier or scenario, are invariably more expensive than endogenous production, the highest and lowest scenarios were included in the graphs for reference. Using these results, it is possible to determine that solely improving the overall process time or having a discount on materials will be insufficient in terms of ultimately being less expensive than the endogenous production. Efforts for future developments should be focused on the rest of the parameters: capital investment, production titer, and bioreactor scale. It is important to note that this analysis did not include recovery yield as a separate variable, since the core calculations of the study were performed using a variable recovery yield and it was therefore not possible to fix its value to a constant in order to show its individual contribution. For this reason, the previous analysis was used to understand its importance and it was concluded that it is critical to CoG/g behavior and its control. These four parameters (including recovery yield) also presented the highest contribution as shown in Figure 2.

For the value of the production titer (Figure 4A) to be less expensive than endogenous production, it should increase less than 2-fold, since the CoG/g for endogenous production is between the 1X and 2X multipliers. This, in turn, compensates for the extreme value used in the previous analysis (10X = 68 g/L) and a 2-fold increase (up to 13.6 g/L) is now a more feasible goal. On the other hand, for the capital cost to be less expensive, it must be decreased further than 0.5X, or to less than half the cost. Moreover, the bioreactor scale must be higher than 250 L of operation in order to be less expensive than its endogenous production at 100 L (a 2.5X increase). From these potential improvements, the production scale and capital decrease can be easily applied to both alternatives, but the production titer represent different complexities depending on the organisms used. As an additional comparison, here is presented the production costs at the mentioned conditions (2-fold increase in production titer and 2.5 fold increase in bioreactor scale, both at the same maximum recovery yield analyzed here—66%), from a recombinant source this is USD $10.91 and from the endogenous source USD $8.64. This comparison further demonstrates the current state of recombinant production, this indicates that additional research is needed to improve recombinant production to fully take advantage of its benefits.

Although it was not directly analyzed in this last section, it is important to constantly consider the overall recovery yield, since it is shown to be the most critical parameter using the data in Table 4. Although it is the most important parameter, it is more challenging to optimize than titer since it involves several unit operations. This will ultimately lead to higher research expenditure in order to increase overall yield. Furthermore, recombinant production has the potential to become less expensive than endogenous if it is possible to generate specific conditions, but ancillary costs need to be considered before actual implementation. Specifically for recombinant production, due to its transgenic nature, it is needed to have proper containment and disposal as well as proper clinical or safety trials depending on the final applications (HA is mostly applied in cosmetic products). On the other hand, endogenous production requires high biosafety facilities as the producing organisms is considered pathogenic and can harm operators and consumers. Analysis of these costs were not included in this work as they were outside of the scope of the analysis, but they have been investigated elsewhere (Puetz and Wurm, 2019), as a reference clinical trials for pharmaceutical proteins have a median of USD $19 million. Overall, the present work has shown specific areas of opportunity for research to guide recombinant production towards a lower production cost and potentially replacing endogenous production of hyaluronic acid.

## Conclusion

The present study has shown the application and contribution of bioprocess modeling and economic analysis towards decision-making for hyaluronic acid production. Given that this study was based on bibliomic data and that the study aims to understand how a diverse array of parameters affect production costs, it was possible to model many different combinations and possible scenarios that would not be possible in real-life situations. However, this process allowed an understanding of the flexibility of CoG/g that will be of value to the future practical implementation of hyaluronic acid production. Moreover, this is the first study to contrast different production strategies which can impact actual processes. It is required to test everything in laboratory conditions, this work has helped to elucidate which research pathways should be further investigated and resources invested.

The results indicate that the most important parameters to consider were recovery yield of the complete bioprocess, production titer at the fermentation stage and bioreactor scale utilized that, in turn, dictates the size of the entire process. Modifications to the current values of these variables, whether increases or decreases, can strongly influence CoG/g and determine the economic viability of a practical implementation. Having a low value of any given variable can cause an increase to above $1,000 USD per gram; alternatively, an optimal value can decrease the cost to below $20 USD *per* gram (i.e., $3.22 USD *per* gram at 10X base titer, 0% discount and 100 L), for all the variables. The lowest cost calculated was $1.53 USD per gram, which was achieved with the simultaneous variation of scale (1,000 L), titer (10X) and recovery yield (66%). Though, for having a recombinant source with a lower production costs than endogenous production, it is needed to increase fermentation titer less than 2 times, which is a feasible objective. This study serves as a framework for future developments and as a guide to decision-making for the next experimental study on the recombinant production of hyaluronic acid.

## Data Availability

The original contributions presented in the study are included in the article/Supplementary Material, further inquiries can be directed to the corresponding author.
