# Supplementary figures and images for: Comparative Economic Analysis Between Endogenous and Recombinant Production of Hyaluronic Acid
Source: Front Bioeng Biotechnol. 2021 Jul 21;9:680278. doi: 10.3389/fbioe.2021.680278 (PMC8334870; doi:10.3389/fbioe.2021.680278)

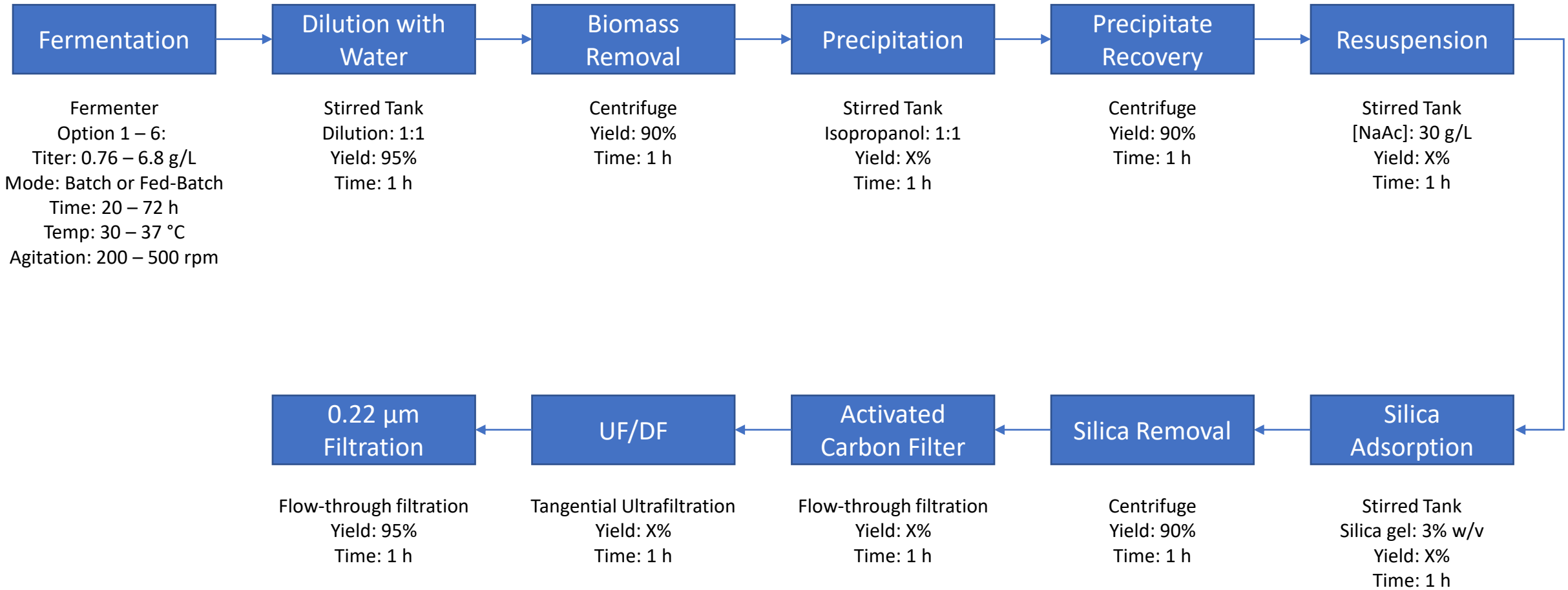

Supplement: Supplementary file 2 [file DataSheet1.PDF]
